# Identification of a GCC transcription factor responding to fruit colour change events in citrus through the transcriptomic analyses of two mutants

**DOI:** 10.1186/1471-2229-10-276

**Published:** 2010-12-15

**Authors:** Gabino Ríos, Miguel A Naranjo, María-Jesús Rodrigo, Enriqueta Alós, Lorenzo Zacarías, Manuel Cercós, Manuel Talón

**Affiliations:** 1 Centro de Genómica, Instituto Valenciano de Investigaciones Agrarias, Carretera Moncada-Náquera km 4.5, 46113 Moncada (Valencia), Spain; 2 Departamento de Ciencia de Alimentos, Instituto de Agroquímica y Tecnología de Alimentos (IATA)-CSIC, Apartado de Correos 73, 46100 Burjassot (Valencia), Spain

## Abstract

**Background:**

External ripening in *Citrus *fruits is morphologically characterized by a colour shift from green to orange due to the degradation of chlorophylls and the accumulation of carotenoid pigments. Although numerous genes coding for enzymes involved in such biochemical pathways have been identified, the molecular control of this process has been scarcely studied. In this work we used the *Citrus clementina *mutants 39B3 and 39E7, showing delayed colour break, to isolate genes potentially related to the regulation of peel ripening and its physiological or biochemical effects.

**Results:**

Pigment analyses revealed different profiles of carotenoid and chlorophyll modification in 39B3 and 39E7 mutants. Flavedo from 39B3 fruits showed an overall delay in carotenoid accumulation and chlorophyll degradation, while the flavedo of 39E7 was devoid of the apocarotenoid β-citraurin among other carotenoid alterations. A *Citrus *microarray containing about 20,000 cDNA fragments was used to identify genes that were differentially expressed during colour change in the flavedo of 39B3 and 39E7 mutants respect to the parental variety. The results highlighted 73 and 90 genes that were respectively up- and down-regulated in both mutants. *CcGCC1 *gene, coding for a GCC type transcriptional factor, was found to be down-regulated. *CcGCC1 *expression was strongly induced at the onset of colour change in the flavedo of parental clementine fruit. Moreover, treatment of fruits with gibberellins, a retardant of external ripening, delayed both colour break and *CcGCC1 *overexpression.

**Conclusions:**

In this work, the citrus fruit ripening mutants 39B3 and 39E7 have been characterized at the phenotypic, biochemical and transcriptomic level. A defective synthesis of the apocarotenoid β-citraurin has been proposed to cause the yellowish colour of fully ripe 39E7 flavedo. The analyses of the mutant transcriptomes revealed that colour change during peel ripening was strongly associated with a major mobilization of mineral elements and with other previously known metabolic and photosynthetic changes. The expression of *CcGCC1 *was associated with peel ripening since *CcGCC1 *down-regulation correlated with a delay in colour break induced by genetic, developmental and hormonal causes.

## Background

Citrus trees produce non-climacteric hesperidium fruits with outstanding agricultural and economic relevance. At the morphological level, citrus fruits consist of an inner edible flesh (endocarp), an intermediate spongy layer (albedo or mesocarp) and an external coloured peel containing pigments and essential oils (flavedo or epicarp). Fruit development in oranges has been divided into three consecutive phases, characterized by a high rate of cell division but slow fruit growth during approximately two months after anthesis (phase I), a second phase of rapid increase in fruit size due to cell enlargement and water accumulation (phase II), and finally a phase of very reduced rate of fruit growth and ripening (phase III) [1].

Citrus fruit maturation shows specific features in flesh and flavedo tissues. Whereas internal maturation in the flesh is accompanied by an increase in the content of solutes and a decrease in acidity, external maturation is typically characterized by a change in colour from green to orange caused by the concomitant catabolism of chlorophylls and the synthesis of carotenoids [2-4]. Under specific environmental conditions, the changes in colour occurring in flavedo may be reversible and are affected by endogenous factors, such as nutrients (sucrose and nitrogen) and phytohormones (gibberellins and ethylene) [5-7]. The biochemical pathways underlying these transformations of pigments have been partially elucidated. Ethylene-induced chlorophyllase activity and gene expression has been negatively related to chlorophyll content suggesting the involvement of the enzyme in colour breakdown of flavedo [8-10]. The characteristic orange colouration of oranges and mandarins is due to the accumulating carotenoids in chromoplasts, particularly oxygenated derivatives (β,β-xanthophylls) and several specific carotenoid cleavage products (apocarotenoids) [11]. Citrus genes coding for enzymes involved in the synthesis and modification of carotenoids have been previously isolated and their evolution during natural and ethylene-induced ripening described [12-16].

Despite such extensive analysis of the physiological and biochemical aspects of fruit external maturation, studies describing induced or natural mutants showing an altered pattern or timing of colour acquisition are scarce yet. Among them, the orange (*Citrus sinensis *L. Osbeck) mutant Pinalate produced yellow-coloured fruits due to an unusually high accumulation of linear carotenes instead of cyclic and oxygenated carotenoids. The mutant also exhibited reduced synthesis of ABA. However, the specific alteration of the carotenoid biosynthesis pathway in Pinalate is currently unknown [17]. The *nan *spontaneous mutation of 'Washington Navel' orange, as formerly characterized in our group, showed an abnormal brown colour in the ripe flavedo caused by a defective catabolism of chlorophylls. Transcript profiling indicated that a *SGR*-like (*STAY-GREEN*) gene was expressed at lower levels in *nan *flavedo, suggesting that *nan *mutation could be associated to a *SGR*-like upstream regulatory factor [18]. Recently, the delay in fruit colouration displayed by the slow-ripening clementine mutant 'Tardivo' (*Citrus clementina *Hort. Ex Tan.) has been associated with altered expression of carotenoid biosynthetic genes and different sensitivity to the exogenous application of ethylene [19].

As part of a mutagenic approach to citrus functional genomics, our group established a collection of near 10,000 independent *Citrus clementina *mutants obtained by fast neutrons bombardment, which were expected to contain genomic deletions in hemizygous dosage. Two of these mutants, called 39B3 and 39E7, were molecularly characterized by array-Comparative Genomic Hybridization for the identification of deleted genes. The structure of 39B3 deletion, determined at the BAC resolution, contained more than 21 identified genes spanning a large genomic region [20]. Phenotypic evaluation for several consecutive years demonstrated that 39B3 and 39E7 mutants have a significant delay in external fruit colour break. In this work we complete the phenotypic characterization and provide the transcriptomic profiling of flavedo from these mutants.

## Results and discussion

### Delay of colour change in 39B3 and 39E7 mutants

**Figure 1:**
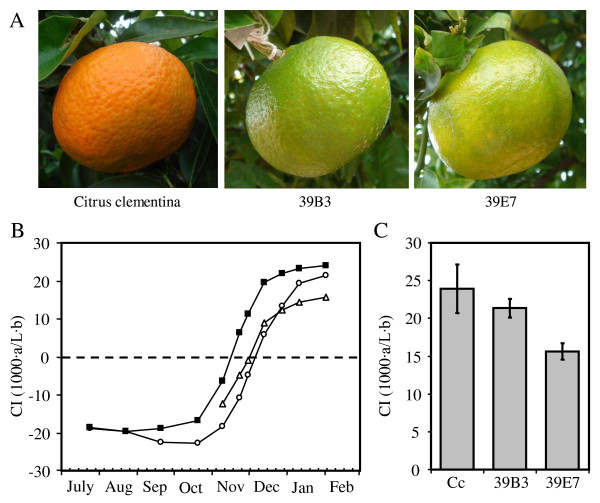
**Phenotype of fruits from 39B3 and 39E7 mutants showing delayed colour change**. (A) External appearance of representative fruits from *Citrus clementina *and 39B3 and 39E7 mutants, photographed at the end of November. The colour index (CI) of flavedo was measured during fruit development and maturation (B) and in fully ripened fruits (C). Clementine (Cc, filled squares), 39B3 (circles) and 39E7 (triangles) mutants. CI = 0, which indicates colour change from green to yellow/orange is shown with a dashed line. Vertical bars in (C) represent standard deviation.

Mutants 39B3 and 39E7 showing delayed fruit colour break for several consecutive years were obtained from a population of near 10,000 *Citrus clementina *plants mutagenized by fast neutrons irradiation. Fruits from 39B3 and 39E7 retained an appreciable greenish colour at the end of November, while fruits from the non-mutagenized parental (for simplification designated as clementine in this work) had already initiated the shift to orange at this time (Figure 1A). A previous structural analysis of the hemizygous genomic deletions found in these mutants reported large DNA lesions containing a high number of genes, but no evidences of overlapping regions in the 39B3 and 39E7 deletions were observed [20]. In order to characterize the nature of colour break alterations affecting these mutants, changes in flavedo colour index (CI) were measured throughout fruit development in both mutants. As shown in Figure 1B, flavedo CI in clementine followed a sigmoid curve shifting from negative (green colour) to positive values (orange colour), approximately at mid November. The pattern of colour change in 39B3 fruits showed a similar behaviour but with a delay of three-four weeks. In 39E7 mutants, however, CI increased at a slower rate (Figure 1B) and reached lower final values than the clementine and 39B3 plants (Figure 1C). These observations suggested that 39B3 and 39E7 mutations affect fruit external ripening in distinct ways; the 39B3 mutation causes a simple delay in flavedo colour change, while the 39E7 mutant is characterized by a reduced rate of colour acquisition leading to an unusual yellowish external appearance after full ripening.

### Chlorophyll and carotenoids accumulation in the mutants

In order to gain a deeper insight into the biochemical alterations affecting 39B3 and 39E7 mutants, total chlorophylls and total and individual carotenoids were determined in flavedo from both mutants and parental fruits at two different developmental stages: in November when colour differences with clementine were more evident (Figure 1A) and in January when all three genotypes had reached the final colouration.

Total chlorophyll pigments were four to five-fold more abundant in 39B3 and 39E7 than in the parental (Table 1), which suggests a slower chlorophyll catabolism or a general delay of maturation. These data were in close agreement with the greenish appearance of mutant fruits in November, while clementine fruits were already changing to orange (Figure 1A). No chlorophylls were however detected two months later, when the three genotypes showed an intense orange (clementine and 39B3) or yellowish (39E7) colouration. Total carotenoids were in 39B3 lower than in clementine in November as expected from the delayed colour break in this mutant, and also in January. On the contrary, 39E7 mutant showed a higher amount of carotenoids in November, but no significant statistical differences were observed with respect to the parental in January.

**Table 1:** Distribution of carotenoids and total carotenoid and chlorophyll content in flavedo from clementine and 39E7 and 39B3 mutants.

	Carotenoids (% of total)					
	
	November			January		
	
	Clementine	39E7	39B3	Clementine	39E7	39B3
*Carotenes*						
Phytoene	9.7 ± 2.8	6.5 ± 0.1	1.6 ± 1.8	10.9 ± 0.8	7.8 ± 2.0	4.6 ± 2.1
Phytofluene	1.9 ± 0.7	1.3 ± 0.1	-	1.8 ± 1.5	1.4 ± 0.5	1.1 ± 0.1
ζ-Carotene	-	-	-	0.2 ± 0.1	-	-
*β,β Carotenoids*						
β-Carotene	tr.	0.4 ± 0.2	0.9 ± 0.1	0.6 ± 0.3	0.5 ± 0.2	0.2 ± 0.1
β-Cryptoxanthin	6.1 ± 0.7	10.3 ± 0.7	14.1 ± 1.2	11.1 ± 2.3	15.1 ± 0.5	13.5 ± 2.0
Zeaxanthin	1.7 ± 0.1	2.1 ± 0.1	2.3 ± 1.3	0.4 ± 0.2	1.1 ± 0.1	0.7 ± 0.1
Anteraxanthin ^a^	5.6 ± 0.1	8.0 ± 0.5	10.6 ± 0.7	7.7 ± 0.7	8.3 ± 0.6	9.7 ± 0.8
E-Violaxanthin	8.1 ± 0.3	17.1 ± 0.3	10.1 ± 1.1	15.8 ± 3.4	17.0 ± 1.3	17.6 ± 1.8
9-Z-Violaxanthin	39.6 ± 3.1	37.7 ± 0.3	57.6 ± 3.7	36.2 ± 4.0	36.3 ± 2.3	36.0 ± 1.8
Neoxanthin	9.6 ± 0.6	1.2 ± 0.3	13.0 ± 0.6	-	-	-
*Apocarotenoids*						
β-Citraurin	3.8 ± 0.2	-	1.7 ± 0.3	4.6 ± 0.7	-	4.0 ± 0.3
8-β-Apocarotenal	0.5 ± 0.2	-		-	-	-
*β,ε Carotenoids*						
α-Cryptoxanthin	-	-	4.0 ± 0.4	-	-	-
Lutein	0.7 ± 0.1	1.5 ± 0.1	5.6 ± 2.6	0.6 ± 0.5	1.5 ± 0.6	1.0 ± 0.1
*Unidentified*	6.4 ± 0.6	5.9 ± 0.2	-	4.2 ± 0.2	3.7 ± 0.7	5.3 ± 1.0

Total carotenoid (μg·g-1 FW)	54.7 ± 3.1	84.4 ± 2.3	38.7 ± 3.2	102.6 ± 11.1	88.9 ± 14.2	66.1 ± 9.1

Chlorophylls (μg·g-1 FW)	11.0 ± 6.6	49.0 ± 11.3	47.5 ± 1.6	-	-	-

Values are mean ± SD of at least three measurements; ^a ^sum of antheraxanthin and mutatoxanthin; - not detected; tr. traces.

The profile of individual carotenoids obtained in the flavedo of clementine essentially coincided with previous reports in this variety, characterized by a reduction in β,ε-carotenoids and neoxanthin and an increase of specific β,β-xanthophylls during ripening [15]. In January, the 39B3 mutant exhibited a carotenoid profile very similar to that of the clementine. However, the pattern of pigment distribution in 39B3 in November differed significantly from the parental, showing lower percentages of phytoene, phytofluene and β-citraurin, and higher amounts of β-carotene, neoxanthin, α-cryptoxanthin and lutein (Table 1), characteristics of chloroplastic tissues, in good agreement with the delayed external colouration in 39B3 fruit. Other β,β-xanthophylls more typical of chromoplastic citrus peel, as β-cryptoxanthin, anteraxanthin and 9-*Z*-violaxanthin, were found in a percentage higher than expected, however the lower amount of total carotenoids in 39B3 indicated a roughly similar absolute accumulation of them in both 39B3 and clementine.

The carotenoid profile of 39E7 mutant showed common features in November and January. In both samples, the absence of the apocarotenoid β-citraurin (C_30_) was associated with a higher accumulation of the xanthophylls β-cryptoxanthin and zeaxanthin. This observation is of special significance because despite the relevant contribution of β-citraurin, a red-orange pigment, to the typical peel colour of oranges and mandarines [11,21], the specific cleavage reaction producing this C_30-_apocarotenoid has not been yet elucidated. The total absence of β-citraurin in fully ripened flavedo of 39E7 mutant suggests that such cleavage reaction could be impaired in this genotype, leading to its distinctive pale yellowish peel. The concomitant increase of β-cryptoxanthin and zeaxanthin in 39E7 might indicate a substrate-product relationship between them and β-citraurin, reinforcing previous suggestions [11,12,21]. Such alteration in the carotenoid biosynthesis pathway corroborates at the biochemical level colour-based observations on the different developmental defects affecting 39B3 and 39E7 mutants. However we cannot rule out the presence of multiple mutations in 39E7 leading to separate effects on colour break delay and carotenoid accumulation. Under this assumption, the observed delay in external colouration could be caused by the same locus in both mutants.

### Differential expression profiling in flavedo

**Figure 2:**
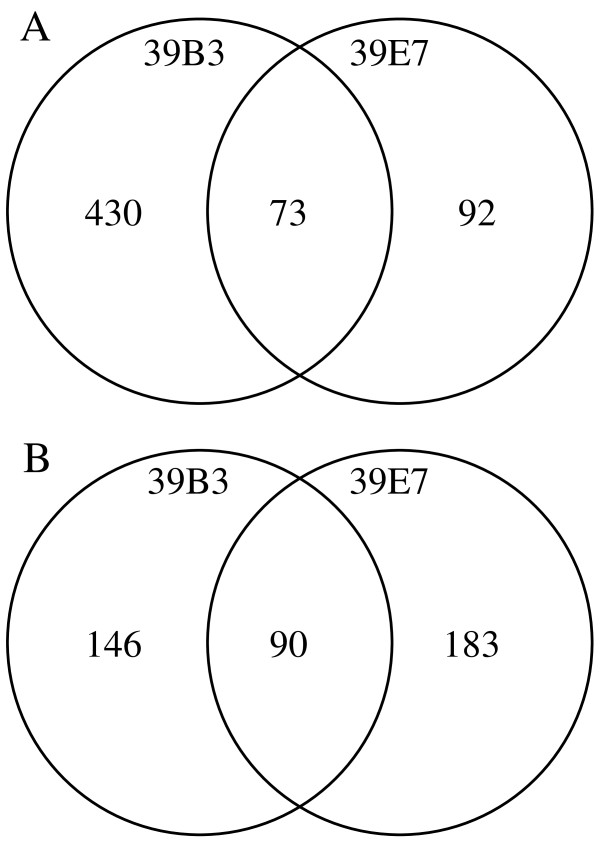
**Transcriptomic analysis of flavedo from 39B3 and 39E7 mutants**. Venn diagrams showing the number of common cDNAs up (A) and down-regulated (B) in flavedo excised from fruits harvested in November from 39B3 and 39E7.

The availability of 39B3 and 39E7 mutants has been exploited to identify major factors involved in regulation of fruit maturation through the transcriptomic analysis of flavedo tissue from these mutants. We took advantage of a citrus cDNA microarray previously described [22] to perform large scale hybridization experiments comparing mRNA isolated from green flavedo of both mutants and clementine flavedo undergoing colour break collected the same day. After microarray hybridization and analysis, cDNAs showing a signal intensity more than double or less than half of control, under a P-value threshold of 10^-5^, were considered as differentially expressed genes. Signal ratios and false discovery rates of selected genes have been included as supplementary material in Additional file 1. As shown in Figure 2 from the 503 and 165 cDNAs overexpressed in 39B3 and 39E7 mutants, respectively, 73 were common. Similarly, a relatively high percentage of down-regulated cDNAs were shared by 39B3 and 39E7 flavedos (90 from 236 and 273, respectively). The occurrence of common transcripts confirms the alteration of particular transcriptional programs in both mutants, which could be revealed by data mining of these coincident clones. None of the 90 cDNAs that were found to be simultaneously down-regulated in both mutants were coincident with the known deleted genes of 39B3 and 39E7. Therefore, they are not expected to reduce their expression as a consequence of their occurrence in a genomic deletion. However, additional deletions to those reported in the published structural characterization of the 39B3 and 39E7 hemizygous deletions [20] might occur in the genome of these mutants and consequently we cannot elucidate whether or not a certain down-regulated gene is included in a deleted fragment.

Functional classification of cDNAs differentially expressed in both 39B3 and 39E7 mutants highlighted major biochemical features underlying peel colour progression (Table 2 and 3). Thus, "photosynthesis" was one of the pivotal enriched categories in the mutants due to the presence in flavedo of photosynthetically active green chloroplasts. Several cDNAs coding for proteins involved in light harvesting, photosynthetic electron transfer chain, Calvin cycle and chlorophyll biosynthesis were overexpressed in mutant samples (Table 2), while no "photosynthesis" category could be created in the list of underexpressed cDNAs (Table 3).

**Table 2:** Genes up-regulated during colour change in the flavedo of both 39B3 and 39E7 mutant fruit.

GenBank	EST	Description	GenBank	EST	Description
**Photosyntesis**			**Transport**		
CX296851	C05803E06	Sedoheptulose-bisphosphatase	CX296724	C05802B02	ChaC-like family protein-like
FC868525	C31001E04	Sedoheptulose-bisphosphatase	FC924529	C31807B06	Predicted protein
FC875746	C31301D05	LHCII type III chlorophyll a/b binding protein	CX305822	C18010F11	T15F16.6 protein
FC923644	C31707D09	LHCII type III chlorophyll a/b binding protein	CX297083	C05808E01	ChaC-like family protein-like
FC875435	C31403H07	Oxygen evolving enhancer protein 1	FC923446	C31705B04	At4g31290
FC920419	C32008B06	Chlorophyll a/b-binding protein CP24	FC874940	C31502E08	Putative mitochondrial dicarboxylate carrier protein
CX287330	C01008D03	Ultraviolet-B-repressible protein	DY300689	KN0AAQ10YP18	Putative cation transporter
FC868970	C31007B05	Photosystem II protein psbY-2	**Response to pathogens**		
CX288326	C01019C02	Chlorophyll a/b binding protein CP29.3			
CX288182	C01017F05	Photosystem I reaction center subunit VI	DY279218	IC0AAA48DB11	Putative thaumatin-like protein
CX287508	C01010C11	Chlorophyll a/b binding protein 8	FC875060	C31503G11	Disease resistance protein putative
CX295423	C05072A10	Photosystem I subunit X	**Calcium binding**		
CX296317	C05138G04	Photosystem I subunit XI			
CX304424	C16011F11	Expressed protein	CX297244	C05810C07	Avr9/Cf-9 rapidly elicited protein 20
DY260996	KN0AAP1YE17	Chlorophyllide a oxygenase	DY261949	KN0AAP4YI13	Regulator of gene silencing
**Metabolism**			**ATP binding**		
CX307598	C19009B12	Xyloglucan endotransglucosylase/hydrolase	DY261553	KN0AAP3YE02	F9H16.5 protein
FC875561	C31405C08	Homocysteine S-methyltransferase	**Redox homeostasis**		
FC919748	C08035D05	Syringolide-induced protein 19-1-5			
FC923462	C31705C11	Aminotransferase 2	FC923741	C31708D11	F1N19.7
FC924391	C31805F07	Glycosyl transferase-like protein	**Others**		
CX295258	C05070C12	Gamma-terpinene synthase			
FC919842	C08036D06	Arabidopsis thaliana genomic DNA chromosome 3 TAC clone:K24A2	CX297093	C05808F01	no annotation available
CX292608	C04017E11	Terpene synthase	CX301234	C08007E01	no annotation available
CX290116	C02020F07	Beta-amylase	FC868898	C31006C04	no annotation available
DY272163	IC0AAA30BF05	Neutral invertase like protein	FC924593	C31807H02	no annotation available
**Regulation of transcription**			FC924767	C31809G10	no annotation available
			CX297352	C05811E08	no annotation available
FC875957	C31303G04	Ethylene-responsive element binding protein ERF4	CX296215	C05136C02	Lectin like protein
FC877608	C31603G11	zinc finger (CCCH-type) family protein	CX308197	C20007C05	Arabidopsis thaliana genomic DNA chromosome 5 TAC clone:K17N15
FC923229	C31702D12	YABBY-like transcription factor GRAMINIFOLIA	CX290048	C02019H10	no annotation available
FC923837	C31709D11	Salt-tolerance protein	CX303737	C16002A07	no annotation available
DY273168	IC0AAA33AG03	Putative glycine-rich zinc-finger DNA-binding protein	FC923410	C31704G03	Expressed protein
FC932314	C34207C06	Putative ethylene response factor 5	FC923118	C31701C08	Putative nematode-resistance protein
CX300605	C07012B10	Emb|CAA19725.1	CX299915	C07004A01	Auxin-binding protein ABP19a precursor
DY260986	KN0AAP1YE03	Dehydration-responsive element binding protein 3	CX301008	C08004F12	UVI1
DY261523	KN0AAP3YC17	Contains similarity to ethylene responsive element binding factor	FC931522	C34106A06	no annotation available
**Protein biosynthesis and modification**			DY283810	IC0AAA5CD09	Arabidopsis thaliana genomic DNA chromosome 5 TAC clone:K18I23
			DY261222	KN0AAP2YC12	T17B22.3 protein
FC932340	C34207E11	Translation initiation factor-like protein	CX290835	C02027F11	expressed protein
CX306680	C18016F10	T13D8.8 protein	FC924819	C31810D08	no annotation available
FC875494	C31404E10	Putative RING-H2 finger protein	DY258718	KN0AAI3AG02	no annotation available
DY276175	IC0AAA40BG02	T13D8.8 protein	DY261234	KN0AAP2YD02	AT5g08050/F13G24_250
			DY261435	KN0AAP2YN14	At1g21010

**Table 3:** Genes down-regulated during colour change in the flavedo of both 39B3 and 39E7 mutant fruit.

GenBank	EST	Description	GenBank	EST	Description
**Metabolism**			**Cell wall modification**		
CX287976	C01015D11	Carbonic anhydrase	CX297394	C06001A06	Alpha-expansin 3
CX289383	C02012D12	Alkaline alpha galactosidase I	DY264363	IC0AAA14BD04	Expansin precursor
CX289985	C02019C03	Putative fatty acid elongase	DY267644	IC0AAA22AB05	Putative pectinesterase
CX289992	C02019C10	Putative aldehyde dehydrogenase	DY295146	IC0AAA87BH09	Expansin precursor
CX292422	C04015E09	Valencene synthase	DY270980	KN0AAP8YH13	Putative pectinesterase
CX298153	C06009B08	Cuticle protein	**Electron transport**		
CX299160	C06019E08	Valencene synthase			
FC919684	C08034F10	Limonoid UDP-glucosyltransferase	CX292526	C04016F10	Cytochrome P450 monooxygenase CYP83A
CX304487	C16012D12	3-ketoacyl-CoA synthase	CX293805	C04035G11	Cytochrome P450-like protein
CX305894	C18011E08	HAD superfamily protein involved in N-acetyl-glucosamine catabolism-like	FC921929	C06054A10	Cytochrome P-450-like protein
CX307823	C20002D06	SRG1 protein	FC919490	C08032F02	Cytochrome P450
FC924270	C31804D03	Cinnamoyl CoA reductase	FC874820	C31501C06	CYP82C1p
FC920274	C32006B10	Glucosyl transferase putative; 93894-95315	FC924343	C31805B05	CYP82C1p
FC930126	C34004A11	Beta-ketoacyl-CoA synthase	FC932589	C34210C05	Cytochrome P450 82A3
FC930590	C34009B09	F3H7.17 protein	DY265052	IC0AAA16BA02	Non-photosynthetic ferredoxin precursor
FC932420	C34208D08	3-ketoacyl-CoA synthase	**ATP binding**		
DY265709	IC0AAA18AD10	Cinnamyl-alcohol dehydrogenase 1			
DY268060	IC0AAA23AF08	Anthranilate synthase alpha subunit precursor	CX290765	C02026H12	Salt-induced AAA-Type ATPase
DY276411	IC0AAA41AD01	Fructose 16-biphosphate aldolase 1	CX300783	C08002B12	UPI0000494294; PREDICTED: DEAD (Asp-Glu-Ala-Asp) box polypeptide 48
DY286831	IC0AAA66AF01	Triterpene UDP-glucosyl transferase UGT71G1	FC921067	C32202G02	AT3g50930/F18B3_210
**Regulation of transcription**			**Others**		
CX287481	C01010A07	Similarity to transfactor	CX289110	C02009C04	no annotation available
**Protein modification**			CX289891	C02018C02	no annotation available
			CX292534	C04016G06	no annotation available
CX291784	C04004H05	Dbj|BAA78736.1	CX293032	C04026G07	T6D22.10
CX297891	C06006D01	Prolylcarboxypeptidase-like protein	CX293318	C04030C06	no annotation available
FC931174	C34102C06	T12M4.17 protein	CX293633	C04033H01	AT4g35240/F23E12_200
FC931272	C34103D03	SOS2-like protein kinase	CX298494	C06012G09	2-on-2 hemoglobin
**Transport**			CX299244	C06020D09	no annotation available
			FC921826	C06052H03	DENN (AEX-3) domain-containing protein-like
CX290491	C02024G06	PDR6 ABC transporter	CX300782	C08002B11	no annotation available
CX298347	C06011C03	Nitrate transporter NRT1-2	CX301411	C08009D08	At1g62790
CX298349	C06011C05	T23G18.9	CX301571	C08011C01	no annotation available
CX307567	C19008G08	sulfate transporter identical to sulfate transporter (Arabidopsis thaliana) GI:2130944	FC919388	C08031E06	no annotation available
CX307912	C20003E08	Ferritin-3 chloroplast precursor	FC919585	C08033F04	Nodulin-like protein
CX309058	C21007H09	Metal transport protein	CX305371	C18004G07	no annotation available
FC874907	C31502B11	Aquaporin	CX305882	C18011D07	no annotation available
FC875147	C31504G02	Plasma membrane H+ ATPase	CX305893	C18011E07	no annotation available
FC924175	C31803D02	Sugar transporter-like protein	CX309162	C18021D09	no annotation available
FC930103	C34003G11	Integral membrane protein putative	CX306953	C18023G08	Nodulin-like protein
FC931689	C34107H08	Putative sulfate transporter ATST1	FC924238	C31804A05	no annotation available
DY279356	IC0AAA49AG01	Nitrate transporter NRT1-5	FC921148	C32101F10	Emb|CAB71107.1
DY280267	IC0AAA50DA03	F10K1.26 protein	FC921343	C32103H04	Nodulin-like protein
DY281465	IC0AAA54AA12	Zinc transporter protein ZIP1	FC930621	C34009E05	no annotation available
DY284165	IC0AAA60CE05	Zinc transporter 4 chloroplast precursor	FC931278	C34103D09	At5g02580
DY260609	KN0AAP13YB08	Arabidopsis thaliana genomic DNA chromosome 5 P1 clone:MUF9	FC930770	C34108H02	Putative embryo-abundant protein
**Response to pathogens**			DY267109	IC0AAA20CC02	no annotation available
			DY283754	IC0AAA5BG06	Expressed protein
CX293128	C04028A01	Major allergen Pru ar 1	DY286094	IC0AAA64CB07	no annotation available
CX295757	C05075E12	HcrVf1 protein	DY260627	KN0AAP13YC02	Flowering promoting factor-like 1
CX297392	C06001A04	Pathogenesis-related protein 10			
FC923487	C31705F01	Thaumatin-like protein isoform 2			

Similarly, known biochemical and physiological features of citrus fruit flavedo at an advanced maturation stage, such as substitution and accumulation of secondary metabolites and cell wall degradation properly correlated with the enrichment and large size of the functional category "metabolism" and to a lesser extend with the category of "cell wall modification" (Table 2 and 3). For example, a valencene synthase responsible for the accumulation of valencene, an important sesquiterpene in the aroma of ripened citrus fruits [23], is catalogued as a down-regulated gene (Table 3). Conversely, a γ-terpinene synthase, involved in the biosynthesis of the monoterpene γ-terpinene in immature green fruits [24], is in the list of up-regulated genes (Table 2).

Interestingly, the transcriptomic study revealed that colour change appears to be also highly dependent upon a major transport activity. The most striking and novel observation in this regard was the high number of putative transporters of mineral elements and metals included in the functional category of "transport" that were down-regulated in the green flavedo of both mutants (Table 3). Thus, several sulfate and nitrate transporters, including a membrane transporter *NRT1*.2 implicated in chloride homeostasis [25], generic metal membrane transporters and specific zinc transporters were common in this category, suggesting that the mobilization of mineral elements such as sulfur, nitrogen, chloride, zinc and other metals may play a relevant role in flavedo ripening. The presence of a gene coding for a ferritin-like protein in the listing of down-regulated cDNAs ([GenBank:CX307912]; Table 3) may exemplify the relevance of these transporters in the colour-break flavedo. Plant ferritins have been described as chloroplastic and mitochondrial proteins involved in Fe(II) oxidation and Fe(III) storage, protecting the cells from the oxidative damage caused by reactive oxygen species produced by free iron [26,27]. For instance, limited iron availability in *Chlamydomonas reinhardtii *has been postulated to induce ferritin coding genes in order to buffer iron released by the degradation of photosystem I (PSI), an important sink for this metal [28]. Similarly, a related ferritin-like gene, up-regulated during leaf senescence in *Brassica napus*, has been proposed to be involved in mobilization of iron from senescing cells to developing organs, where the metal is highly required [29]. Thus, ferritin accumulation in clementine flavedo tissue undergoing colour break may apparently contribute to the sequestering and recycling of iron molecules released during the degradation of photosystems and light-harvesting complexes, at the transition from chloroplast to chromoplast. The membrane transporters listed in Table 3 could initiate subsequent mobilization of the sequestered iron and maybe other metals and mineral elements to the cells requiring them.

The category of "transport" was also enriched with cDNAs coding for other several kinds of transporters including ABC transporters, sugar and protein transporters, aquaporin, H^+^-ATPases and other unidentified membrane transporters associated with the green stage of the flavedo (Table 3).

### A MYB-related transcription factor down-regulated in 39B3 and 39E7

Whereas nine different cDNAs coding for transcriptional regulators, including three ethylene response factors, were up-regulated in 39B3 and 39E7 (Table 2), only one was down-regulated in both mutants ([GenBank:CX287481]; Table 3). This transcription factor belongs to a subgroup of the GARP (***G***OLDEN2, ***AR***R-B and ***P***sr1) subfamily of MYB-related proteins containing a coiled-coil domain, which has been recently designated GCC (***G***ARP and ***c***oiled-***c***oil) [30,31]. Consequently, we named the protein deduced from this cDNA CcGCC1 (for *Citrus clementina ****GCC***). This gene was not found in a previous genomic approach to identify deleted genes in 39B3 and 39E7 mutants [20], and hence no gene dosage effects are expected to contribute to lower its expression in the mutants.

**Figure 3:**
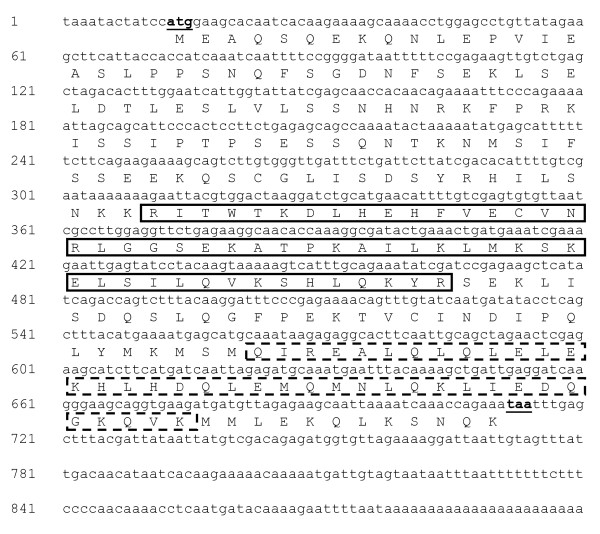
**Full-length cDNA and deduced protein of *CcGCC1 *gene**. Start and stop codons are underlined. In the deduced protein, continuous and dashed lines surround, respectively, the GARP and coiled-coil domains.

The partial sequence of *CcGCC1 *cDNA annotated in clone [GenBank:CX287481] was completed by sequencing its 3' end. The resulting nucleotide and amino acid sequences are shown in Figure 3. Database similarity search by BLASTP analysis [32] of the 233 residues long protein deduced from the cDNA confirmed a high similarity to other members of the GCC subgroup. We used the SMART [33] and COILS [34] applications to localize the GARP DNA-binding and the coiled-coil domains respectively, which are highlighted in Figure 3.

**Figure 4:**
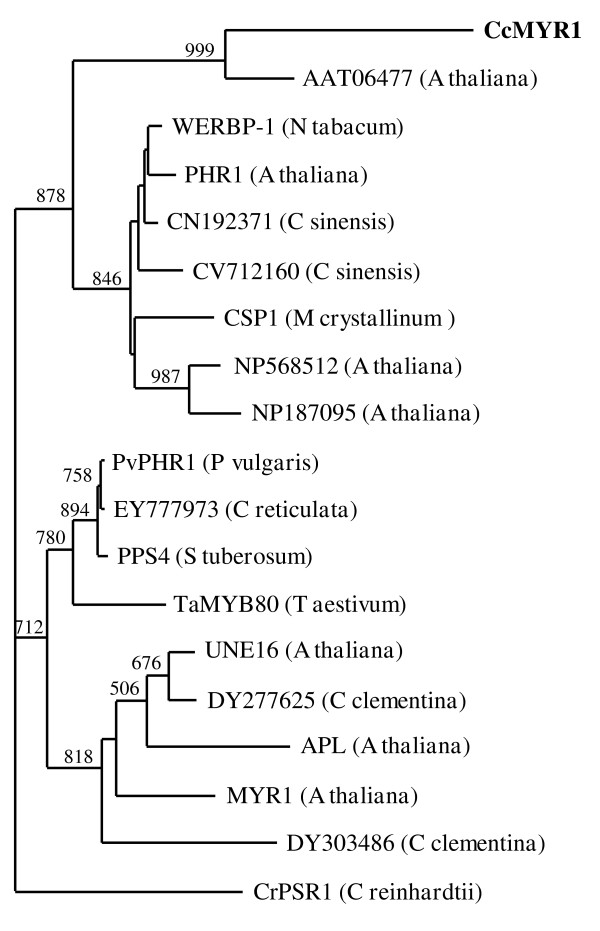
**Phylogenetic analysis of GCC like amino acid sequences**. Ninety residues long fragments of GARP and coiled-coil domains of nineteen proteins were aligned using ClustalW2 program and analyzed as described in Methods. Proteins selected were [GenBank:AAT06477] (At5g06800 from *Arabidopsis thaliana*), [GenBank:BAA75684] (WERBP-1 from *Nicotiana tabacum*), [REFSEQ:NP_568512] (At5g29000 from *Arabidopsis thaliana*), [REFSEQ:NP_194590] (PHR1 from *Arabidopsis thaliana*), [REFSEQ:NP_187095] (At3g04450 from *Arabidopsis thaliana*), [GenBank:AAF32350] (CSP1 from *Mesembryanthemum crystallinum*), [GenBank:AAU06822] (TaMYB80 from *Triticum aestivum *), [GenBank:BAE46413] (PPS4 from *Solanum tuberosum*), [GenBank:ACD13206] (PvPHR1 from *Phaseolus vulgaris*), [GenBank:AAD55941] (CrPSR1 from *Chlamydomonas reinhardtii*), [REFSEQ:NP_974798] (MYR1 from *Arabidopsis thaliana*), [REFSEQ:NP_567408] (UNE16 from *Arabidopsis thaliana*), [REFSEQ:NP_849905] (APL from *Arabidopsis thaliana*), [GenBank:CV712160] (*Citrus sinensis*), [GenBank:CN192371] (*Citrus sinensis*), [GenBank:EY777973] (*Citrus reticulata*), [GenBank:DY303486] (*Citrus clementina*) and [GenBank:DY277625] (*Citrus clementina*). Clementine CcGCC1 protein is indicated in bold. Bootstrap values higher than 500 (of 1000 samples) are shown for each node.

In order to compare *CcGCC1 *and other related citrus ESTs with known members of this GCC subgroup, we selected a 90 amino acids long fragment fusing GARP and coiled-coil domains of CcGCC1 and several homologous proteins and translated ESTs [35-41]. The phylogenetic tree of these proteins showed two major groups with CcGCC1 clustered with PHR1 from *Arabidopsis thaliana*, a protein involved in phosphate starvation signalling (Figure 4). The closest homolog to CcGCC1 among those polypeptides was [GenBank:AAT06477], coded by At5g06800 gene from *Arabidopsis*. Interestingly, a search into the AtGenExpress database containing microarray expression data of *Arabidopsis *genes revealed that At5g06800 is mostly expressed in tissues lacking chloroplasts such as roots and to a lesser extent pollen and flower organs [42].

### *CcGCC1 *expression correlates with colour change processes

**Figure 5:**
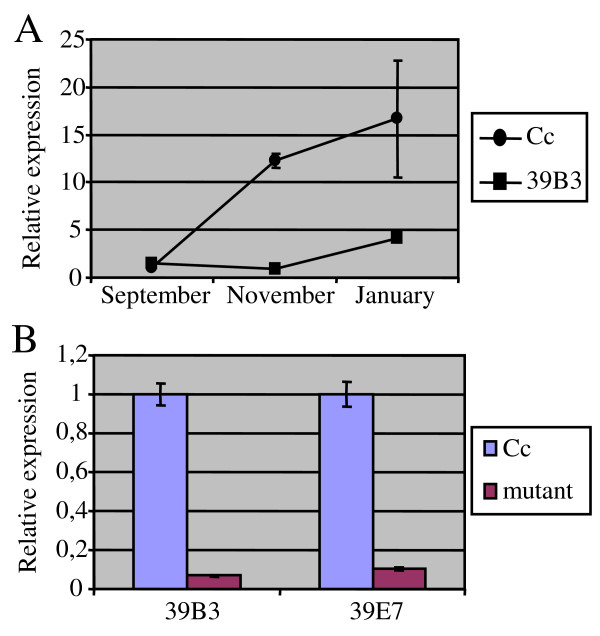
**Expression analysis of *CcGCC1 *gene in flavedo during colour change**. (A) Relative expression level of *CcGCC1 *gene in flavedo from fruits of clementine (Cc) and 39B3 mutant at three developmental stages. In September, both clementine and 39B3 show a green immature flavedo; in November, flavedo from clementine exhibits orange and yellowish colours while 39B3 flavedo is still green; and finally, in January, both genotypes show fully orange coloured flavedos. (B) Relative expression level of *CcGCC1 *gene in 39B3 and 39E7 flavedos excised from fruits harvested in November. Vertical bars represent standard deviation.

In order to investigate the time-dependent expression of *CcGCC1 *during fruit external maturation in clementine, flavedo tissues collected before (September), during (November) and after fruit colour break (January), were subject to RNA extraction and quantitative RT-PCR with *CcGCC1 *specific primers. Figure 5A shows that the expression level of *CcGCC1 *in clementine increased about 15-fold during flavedo ripening, while 39B3 mutant maintained low expression levels in November and only experienced a slight increase in January. A similar change on *CcGCC1 *expression was observed in 39E7 mutant when samples harvested in November were assayed (Figure 5B). These results confirmed that *CcGCC1 *gene expression was induced during colour break of clementine fruits whereas mutants 39B3 and 39E7 affected in the rate of colour break were unable to properly express the gene.

**Figure 6:**
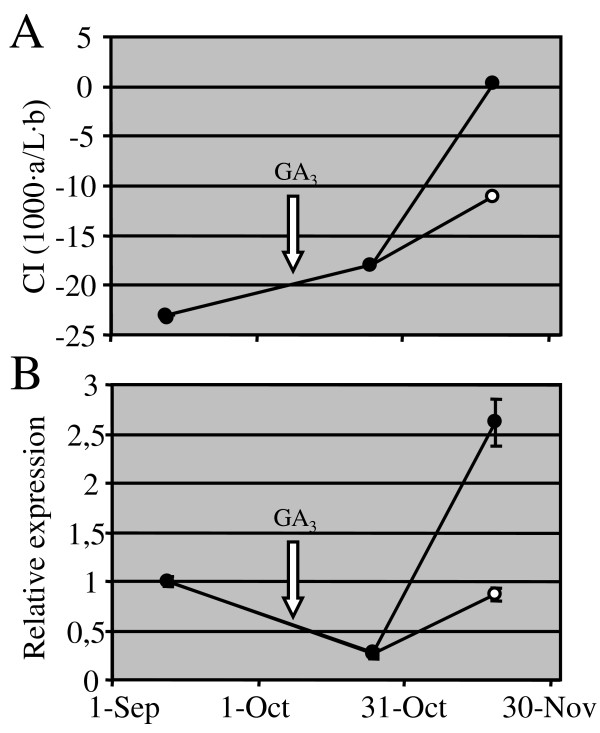
**Effect of gibberellin application on flavedo colour change and *CcGCC1 *expression**. (A) Colour index (CI) of flavedo from GA_3_-treated (empty circles) and untreated (filled circles) fruits of clementine. (B) Relative expression of *CcGCC1 *gene in these samples. Successive applications of GA_3 _were periodically made and the arrow is labelling the first GA_3 _treatment. Vertical bars represent standard deviation.

To determine if the expression of *CcGCC1 *gene was also responsive to other factors modulating colour change, a further experiment using external applications of gibberellins was performed (Figure 6). Gibberellins (GA) operate as colour change retardants during fruit external maturation since GA application on green flavedo causes a significant delay in colour break [14,15]. Forty-two days after the first application, fruits treated periodically with gibberellin A_3 _showed a delay of about 10 colour units with respect to untreated fruits (Figure 6A). Interestingly, the GA-dependent retard in peel colour was accompanied by a parallel delay in *CcGCC1 *induction (Figure 6B). These results indicate that *CcGCC1 *also responds to the GA-dependent pathway regulating flavedo ripening and taken together with the previous observations suggest the participation of CcGCC1 in a regulatory pathway acting in parallel or subsequently to colour break processes.

## Conclusions

In this work, the citrus mutants 39B3 and 39E7 showing a delay in fruit colour change have been phenotypically characterized and used to identify through the analyses of their transcriptomes about 160 genes that were directly related to peel ripening. The results indicated that the 39B3 genotype exhibits a simple delay in the rate of flavedo colouration, while 39E7 shows an additional altered pattern of carotenoid accumulation. We postulate that the yellowish colour of fully ripe 39E7 flavedo was due to a defective synthesis or accumulation of β-citraurin. Analyses of differentially expressed genes revealed that colour change during peel ripening was strongly associated with a major mobilization of mineral elements and other previously known metabolic and photosynthetic changes. Transcriptomic data also showed that expression of *CcGCC1 *gene coding for a transcription factor containing GARP and coiled-coil domains, was strongly down-regulated in flavedo tissue of both mutants. Application of gibberellin to green fruits postponed colour break and abolish the induction of *CcGCC1 *expression. Taken together the results indicated that *CcGCC1 *down-regulation correlated with a delay in colour break induced by genetic, developmental and hormonal cues.

## Methods

### Plant material

About 7 years-old clementine trees (*Citrus clementina *Hort. Ex Tan. cv. clemenules) were grown at the Instituto Valenciano de Investigaciones Agrarias (IVIA) under standard agricultural practices. The 39B3 and 39E7 mutants were obtained by fast neutrons irradiation of clemenules buds [43].

### GA_3 _treatment

Individually labelled fruits were periodically treated on-tree with 60 mg/L gibberellin A_3 _(GA_3_) (Sigma). In each treatment, fruits on four adult trees were sprayed every 3 days from October 7 (189 days after anthesis) to November 18 (231 days after anthesis). After colour index determination (see below), flavedo tissue from treated and untreated trees was collected at three different dates: September 12 (previous to GA_3 _treatment), October 25 and November 18 [15].

### Colour index determination

The L, *a*, and *b *Hunter lab parameters of the colour system were measured on the flavedo surface with a Minolta CR-200 chromameter. The values presented are the results of the 1000 *a*/L*b *transformation that results in negative and positive values for the green and orange colours, respectively, in citrus fruit [44]. In this transformation, the zero value coincides with the midpoint of the colour break period. Eight and twenty fruits were measured per sample for the colour change curve and the GA experiment respectively.

### Extraction and quantification of chlorophylls and carotenoids

Flavedo pigments were extracted as previously described [17]. Briefly, frozen ground material (500 mg) of flavedo was extracted with a mixture of methanol and 50 mM Tris-HCl buffer (pH 7.5) containing 1 M NaCl and partitioned against chloroform until plant material was uncoloured. The chlorophyll (*a*+*b*) content was determined by measuring the absorbance of the extracts at 644 nm and 662 nm and calculated according to the Smith and Benitez equations [45]. After chlorophylls measurement, the pigment ethereal solution was dried and saponified using a KOH methanolic solution. The carotenoids were subsequently re-extracted with diethyl ether. Extracts were dried under N_2 _and kept at -20 ºC until HPLC analysis. Prior to HPLC analysis, carotenoid extracts were dissolved in acetone and incubated overnight at -20ºC to precipitate sterols that could interfere in the carotenoid analysis and subsequently dried under N_2_.

Carotenoid composition of each sample was analyzed by HPLC with a Waters liquid chromatography system equipped with a 600E pump and a model 996 photodiode array detector, and Empower software (Waters). A C30 carotenoid column (250 × 4.6 mm, 5 μm) coupled to a C30 guard column (20 × 4.0 mm, 5 μm) (YMC Europe GMBH) was used. Samples were prepared for HPLC by dissolving the dried carotenoid extracts in CHCl_3_: MeOH: acetone (3:2:1, v:v:v). A ternary gradient elution with MeOH, water and methyl *tert*-butyl ether (MTBE) was used for carotenoid separation reported in previous works [17,46]. Briefly, the initial solvent composition consisted of 90% MeOH, 5% water and 5% MTBE. The solvent composition changed in a linear fashion to 95% MeOH and 5% MTBE at 12 min. During the next 8 min the solvent composition was changed to 86% MeOH and 14% MTBE. After reaching this concentration the solvent was gradually changed to 75% MeOH and 25% MTBE at 30 min. Final composition was reached at 50 min and consisted of 50% MeOH and 50% MTBE. Initial conditions were re-established in 2 min and re-equilibrated for 15 min before next injection. The flow rate was 1 mL/min, column temperature was set to 25°C and the injection volume was 20 μL. The photodiode array detector was set to scan from 250 to 540 nm, and for each elution a Maxplot chromatogram was obtained, which plots each carotenoid peak at its corresponding maximum absorbance wavelength. Carotenoids were identified by comparison of the spectra and retention time with those of authentic standards, when available, or by matching the observed versus literature spectral data and retention time under identical chromatographic conditions [12,46,47]. The carotenoid peaks were integrated at their individual maxima wavelength and their content were calculated using calibration curves of β-apo-8'-carotenal (a gift from Hoffman-LaRoche) for apo-8'-carotenal and β-citraurin, β-cryptoxanthin (Extrasynthese) for α- and β-cryptoxanthin, lutein (Sigma) for lutein, neoxanthin, violaxanthin isomers and mutatoxanthin, zeaxanthin (Extrasynthese) for zeaxanthin and antheraxanthin, and β-carotene (Sigma). Standards of phytoene, phytofluene and ζ-carotene for quantification were obtained from flavedo extracts of Pinalate fruits, which accumulate large amounts of these compounds [17], and afterward purified by TLC.

Samples were extracted at least twice and each analytical determination was replicated at least once. All operations were carried out on ice under dim light to prevent photodegradation, isomerisations and structural changes of carotenoids.

### Expression profiling

Total RNA was isolated from flavedo of clementine and mutant fruits collected in November, using RNeasy Plant Mini Kit (Qiagen). The transcripts present in 1.5 μg of total RNA were reverse-transcribed, amplified and labelled with the Amino Allyl MessageAmp™II aRNA Amplification kit (Ambion), following the manufacturer's instructions. Cy3 and Cy5 fluorescent dyes coupled to the aRNA were obtained from the CyDye™Post-Labeling Reactive Dye Pack (Amersham). Purified Cy5 and Cy3 labelled probes (200 pmol each) were combined, diluted with water to a final volume of 9 μL, and fragmented using the RNA Fragmentation Reagents (Ambion). Fragmented samples were heat-denatured for 2 min at 80 ºC, mixed with 50 μL of pre-heated hybridization buffer (5 × SSC, 50% formamide, 0.1% SDS, 0.1 mg/mL salmon sperm DNA) and applied to the microarray slide prehybridized in 5 × SSC, 0.1% SDS, 1% BSA, for at least 1 h at 42 ºC. We employed the 20 K *Citrus *cDNA microarrays containing 21240 EST generated by the Spanish Citrus Functional Genomics Project [22,48,49]. Three biological replicates of each mutant were compared to three replicates of control in a dye-swap experiment requiring six slides per mutant.

Hybridization was performed overnight at 42 ºC. After hybridization, slides were washed 5 min twice at 42 ºC in 2 × SSC, 0.1% SDS followed by two washes at room temperature for 5 min in 0.1 × SSC, 0.1% SDS, then by 5 washes at room temperature for 1 min in 0.1 × SSC and rinsed briefly in 0.01 × SSC before drying by centrifugation at 300 rpm 5 min.

Arrays were scanned at 5 μm. Cy3 and Cy5 fluorescence intensity was recorded by using a ScanArray Gx (Perkin Elmer). The resulting images were overlaid and spots identified by the ScanArray Express program (Perkin Elmer). Spot quality was first measured by the signal-to-background method with parameters lower limit (200) and multiplier (2), and subsequently confirmed by visual test. Data analysis was performed using the Limma package from the R statistical computing software [50]. A mutant/wild type signal higher than 2 or lower than 0.5, with a P-value not higher than 10^-5 ^were the cut-off values for positive EST identification. The 39B3 and 39E7 microarray experiments have been loaded into the ArrayExpress database under accessions E-MEXP-2638 and E-MEXP-2641, respectively.

### Quantitative RT-PCR

Total RNA was isolated from excised flavedo using RNeasy Plant Mini Kit (Qiagen). RNA concentration was determined by a fluorometric assay with the RiboGreen dye (Molecular Probes) following the manufacturer's instructions. Five μg of total RNA was reverse transcribed with the SuperScript III First-Strand Synthesis System for RT-PCR (Invitrogen) in a total volume of 20 μL. Two μL of a 20 times diluted first-strand cDNA was used for each amplification reaction. Quantitative real-time PCR was performed on a LightCycler 2.0 instrument (Roche), using the LightCycler FastStart DNA MasterPLUS SYBR Green I kit (Roche). Reaction composition and conditions followed manufacturer's instructions. The primers employed were 5'-CCGAGAAGTTGTCTGAGCTAGA-3' and 5'-CCCACAAGACTGCTTTTCTTCT-3', which amplified a fragment of 164 base pairs on a cDNA template. Cycling protocol consisted of 10 min at 95°C for pre-incubation, then 40 cycles of 10 sec at 95°C for denaturation, 10 sec at 60°C for annealing and 10 sec at 72°C for extension. Fluorescent intensity data were acquired during the extension time. Specificity of the PCR reaction was assessed by the presence of a single peak in the dissociation curve after the amplification and through size estimation of the amplified product by gel electrophoresis. For expression measurements, we used the absolute quantification analysis from the LightCycler Software 4.0 package (Roche), and calculated expression levels relative to values of a reference sample. Reference sample was flavedo from the parental clementine in Figure 5 and flavedo tissue before GA application in Figure 6. Results were the average of 3 independent biological replicates repeated twice.

### Phylogenetic analysis

The fused sequences of the GARP and coiled-coil domains of CcGCC1 and other 18 proteins obtained or deduced from databases were aligned with the ClustalW2 program [51]. Phylogenetic analysis was performed using programs from the PHYLIP.

group, PHYLogeny Inference Package, Version 3.6 [52,53]. A distance matrix was computed according to the Dayhoff PAM model by the program Protdist and then it was used as input for the program Neighbor, where the Neighbor-joining method of clustering was selected. A bootstrap analysis based on 1000 replicates was performed. CrPSR1 from *Chlamydomonas reinhardtii *was defined as the outgroup species.

## Authors' contributions

GR carried out transcriptomic and sequence analysis and drafted the manuscript. MAN carried out real-time PCR analyses. MJR and LZ performed pigments measurements and contributed to draft the manuscript. EA and MC designed and made the GA experiment. MT conceived the study and assisted in the drafting of the manuscript. All the authors read and approved the final manuscript.

## Supplementary Material

Additional file 1 **Supplementary material**. Log2 signal ratio (M) and P value of ESTs shown in Table 2 and 3 after microarray hybridization experiments. Click here for file
